# Evaluation of the Antinociceptive Activity and Acute Oral Toxicity of Standardized Ethanolic Extract of the Rhizome of *Curcuma xanthorrhiza* Roxb

**DOI:** 10.3390/molecules15042925

**Published:** 2010-04-22

**Authors:** Sutha Devaraj, Azadeh Sabetghadam Esfahani, Sabariah Ismail, Surash Ramanathan, Mun Fei Yam

**Affiliations:** 1 Centre for Drug Research, Universiti Sains Malaysia, 11800, Pulau Pinang, Malaysia; 2 School of Pharmaceutical Sciences, Universiti Sains Malaysia, 11800, Pulau Pinang, Malaysia

**Keywords:** analgesic, acute oral toxicity, *Curcuma xanthorrhiza*, peripheral, standardized

## Abstract

Ethanolic extract of *Curcuma xanthorrhiza* was used to evaluate the analgesic and toxicity effects *in vivo*. The extract was standardized using GC-MS, which showed that 1 mg of *Curcuma xanthorrhiza* ethanolic extract contains 0.1238 mg of xanthorrhizol. The analgesic activity was studied in rats using three different models, namely the hot plate test, tail flick test and formalin-induced pain test. The acute oral toxicity was examined by the oral administration of standardized *Curcuma xanthorrhiza* ethanolic extract in mice at doses ranging from 300–5,000 mg/kg and observation for 14 days. Standardized *Curcuma xanthorrhiza* ethanolic extract did not show significant analgesic effect in the hot plate and tail flick tests. However, in the formalin-induced pain test, *Curcuma xanthorrhiza* ethanolic extract significantly (P < 0.05) suppressed the paw licking time of rats in both early and late phases at doses 200 and 400 mg/kg of the extract, respectively. In the acute oral toxicity study, *Curcuma xanthorrhiza* ethanolic extract did not show any toxic effects in mice at 5 g/kg. These experimental results suggest that the standardized *Curcuma xanthorrhiza* ethanolic extract showed peripheral and central antinociceptive activity associated with neurogenic pain as well as a relative absence of toxic effects which could compromise the medicinal use of this plant in folk medicine.

## 1. Introduction

*Curcuma xanthorrhiza* Roxb. is a member of the ginger family (Zingiberaceae) and a native Indonesian plant. It is grown in Thailand, Philippines, Sri Lanka and Malaysia. It is commonly known in Malaysia as ‘Temu Lawak’ [1]. *Curcuma xanthorrhiza* is a low growing plant with a root (rhizome) which is similar to ginger, with an aromatic, pungent odor and bitter taste. In traditional medicine *Curcuma xanthorrhiza* is reported to be useful for the treatment of hepatitis, liver complaints, diabetes, rheumatism, anticancer, hypertension and heart disorders. *Curcuma xanthorrhiza* has also shown antidiuretic, anti-inflammatory, anti-oxidant, anti-hypertensive, anti-rheumatic, anti-hepatotoxic, anti-dysmenorrheal, anti-spasmodic, anti-leucorrhoea, anti-bacterial and antifungal effects [1]. It reduces cholesterol, treats constipation, migraines and increases flow of milk during breast feeding. The *Curcuma xanthorrhiza* aqueous extract revealed significant hepatoprotective activity against β-D-galactosamine induced liver damage [2]. Ozaki has reported on the anti-inflammatory activity of the *Curcuma xanthorrhiza* methanolic extract [3].

The traditional benefits of *Curcuma xanthorrhiza* were further supported by the isolation and identification of several active chemical constituents, including xanthorrhizol, curcumin and few volatile substances. Xanthorrhizol, the major component of the essential oil of *Curcuma xanthorrhiza*, is a bisabolane-type sesquiterpenoid. This compound makes up nearly 46.3% of the total components of the essential oil obtained through hydrodistillation [4]. Xanthorrhizol was investigated for its antibacterial [5] and anticandidal activity [6]. Lim *et al*, [7] have reported on the anti-inflammatory effects of xanthorrhizol against neuronal cells. Curcumin belongs to the phenol group and comprises almost 1 to 2% in ethyl acetate fraction of *Curcuma xanthorrhiza*. It is the second essential active compound found in this plant [8]. Curcumin has been shown to have a powerful anti-inflammatory action since it inhibits the mechanisms of arachidonic acid and leukotrine formation [1]. *Curcuma xanthorrhiza* contains various active volatile substances and borneol, which are present in much lower quantities [9]. *Curcuma xanthorrhiza* methanolic extract has shown significant antidiuretic activity [10]. Nevertheless, analgesic and toxicity studies on this plant are very limited and most work was undertaken using *Curcuma xanthorrhiza* methanolic and aqueous extracts [3,10]. Moreover, to date, no work has been done using ethanolic extract preparation for analgesic and toxicity screening in animals. Therefore, this study aims to investigate the acute oral toxicity and antinociceptive activity of the rhizome of *Curcuma xanthorrhiza* ethanolic extracts using the hot plate, tail flick and formalin tests on animal models. 

## 2. Results and Discussion

### 2.1. Standardization of Curcuma xanthorrhiza ethanolic extract

**Figure 1:**
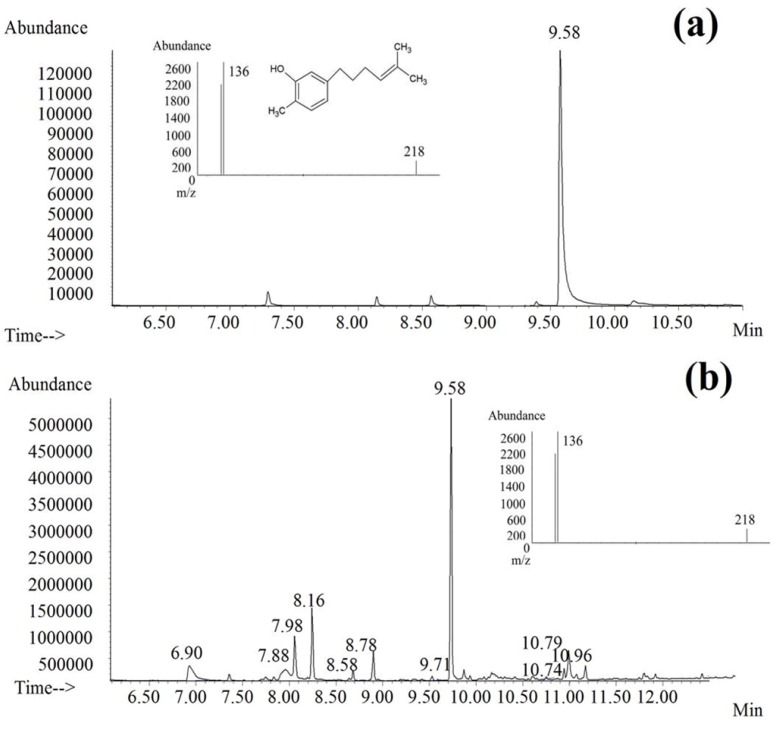
Typical chromatograms from GC-MS analysis of (a) xanthorrhizol and (b) *Curcuma xanthorrhiza* ethanolic extract. Insets: (a) mass spectrum and chemical structure of xanthorrizol; (b) mass spectrum of *Curcuma xanthorrhiza* ethanolic extract with a peak at 9.58 min; Chromatographic conditions: HP-5MS capillary column (30 m × 0.25 mm × 0.25 µm); Injector temperature: 280 °C; Oven temperature: 70 °C–250 °C; Injection volume: 1µL; Flow rate: 0.5 mL/min. Electron impact (EI):70 eV.

GC-MS analysis of *Curcuma xanthorrhiza* ethanolic extract yielded a linear calibration curve for the standard compound, xanthorrhizol, which is the main compound found in this plant. The correlation coefficient was 0.998 for the respective standard curve. Xanthorrhizol was identified and quantified at 9.58 min retention time and in the *Curcuma xanthorrhiza* ethanolic extract as shown in Figure 1. The results revealed that 1 mg of *Curcuma xanthorrhiza* ethanolic extract contains 0.1238 mg of xanthorrhizol.

### 2.2. Phytochemical screening

The qualitative phytochemical screening tests of standardized *Curcuma xanthorrhiza* ethanolic extract revealed that the extract contains reducing sugars, saponins, anthraquinones, flavonoids, terpenoids and cardiac glycosides. Alkaloid, steroid, tannins and phlobatannins were absent in the *Curcuma xanthorrhiza* ethanolic extract, as summarized in Table 1.

**Table 1:** Phytochemical profile of standardized ethanolic extract of *Curcuma xanthorrhiza* rhizome.

Chemical constituents	Tests/Reagents	Results
Reducing sugars	Fehling’s reagent	++
Saponins	Frothing test	+
Alkaloids	Dragendroff’s reagent	-
Flavonoids	Acid-alcohol	+++
Cardiac Glycosides	Keller-Killiani test	+++
Steroids	Acetic anhydride	-
Terpenoids	Sulphuric acid reagent	+++
Tannins	Ferric chloride reagent	-
Phlobatannin	Hydrochloric acid	-
Anthraquinones	Borntrager’s test	++

Key: +++ = abundance; ++ = moderately present; + = present; ± = weakly present; - = absent.

### 2.3. Acute oral toxicity study

Oral administration of standardized *Curcuma xanthorrhiza* ethanolic extract showed no mortality in mice at doses up to 5 g/kg. There were no toxicity signs observed on the skin, fur or eyes of the animals. No noticeable behavioural changes in salivation, sleeping pattern, diarrhea or lethargy were spotted in the treated animals. This result indicates that the *Curcuma xanthorrhiza* ethanolic extract is non-toxic, and safe at 300 mg/kg, 2,000 mg/kg and 5,000 mg/kg. This explains the rational usage of this extract in folk medicine practices.

### 2.4. Hot plate and tail flick tests

The mean latency time of the analgesic effect of standardized *Curcuma xanthorrhiza* on the thermal pain stimuli is summarized in Table 2 and Table 3. 

**Table 2:** The effects of standardized ethanolic extract of *Curcuma xanthorrhiza* (CX) rhizome in the hot plate test.

Groups (Dose)	Latency of nociceptive response (s) ( *n* = 5)				
	0	15	30	45	60 (min)
Control	7.05 ± 0.70	7.44 ± 0.25	8.36 ± 1.01	8.41 ± 1.25	10.32 ± 2.16
CX (100 mg/kg)	8.82 ± 0.70	8.34 ± 1.62	11.95 ± 1.63	13.60 ± 1.71	13.86 ± 0.14
CX (200 mg/kg)	8.82 ± 0.70	10.92 ± 0.80	13.86 ± 2.61	13.28 ± 1.85	13.93 ± 0.86
CX (400 mg/kg)	9.44 ± 0.96	15.59 ± 2.48	12.72 ± 2.48	14.44 ± 2.99	12.52 ± 2.26
Aspirin (300 mg/kg)	8.97 ± 0.89	16.27 ± 2.49	16.85 ± 3.85	14.80 ± 3.39	16.17 ± 5.61
Morphine (5 mg/kg)	18.03 ± 0.55*	22.08 ± 0.65*	22.74 ± 0.76*	10.72 ± 1.67*	8.78 ± 0.92

Data are Mean ± SEM; * p < 0.05, evaluated by one-way analysis of variance (ANOVA) against control group.

**Table 3:** The effects of standardized ethanolic extract of *Curcuma xanthorrhiza* (CX) rhizome in tail flick test.

Groups (Dose)	Latency of nociceptive response (s) ( *n* = 5)				
	0	15	30	45	60 (min)
Control	3.16 ± 0.20	4.19 ± 0.40	3.16 ± 0.21	3.92 ± 0.34	4.71 ± 0.85
CX (100 mg/kg)	2.40 ± 0.39	2.56 ± 0.43	2.63 ± 0.35	3.36 ± 0.26	3.80 ± 0.82
CX (200 mg/kg)	3.34 ± 0.34	3.44 ± 0.62	2.69 ± 0.30	3.50 ± 0.36	3.03 ± 0.43
CX (400 mg/kg)	3.14 ± 0.62	3.23 ± 0.42	3.12 ± 0.43	3.38 ± 0.44	3.95 ± 0.17
Aspirin(300 mg/kg)	2.92 ± 0.20	2.52 ± 0.21	3.42 ± 0.32	3.37 ± 0.35	4.21 ± 0.46
Morphine (5 mg/kg)	10.00 ± 0.00*	8.65 ± 0.89*	8.63 ± 0.78*	7.09 ± 1.23	7.19 ± 1.28

Data are Mean ± SEM; * p < 0.05, evaluated by one-way analysis of variance (ANOVA) against control group.

The ethanolic extract of *Curcuma xanthorrhiza* did not show significant antinociceptive effects on pain induced by hot plate and tail flick in rats compared to the control at any of the tested doses (100, 200 and 400 mg/kg). The hot plate test involves the supraspinal nociceptive system [11], whereas the tail flick nociceptive response is transferred through the spinal dorsal horn in the central analgesic system [12]. In contrast, morphine exerted a significant increase (p < 0.05) in the response time in the hot plate and tail flick experiments, respectively, since it is a centrally acting narcotic drug [13]. On contrary, in this experiment, aspirin is inactive since it is a peripherally acting non-steroidal anti-inflammatory drug (NSAID) [14]. 

### 2.5. Formalin induced pain test

**Figure 2:**
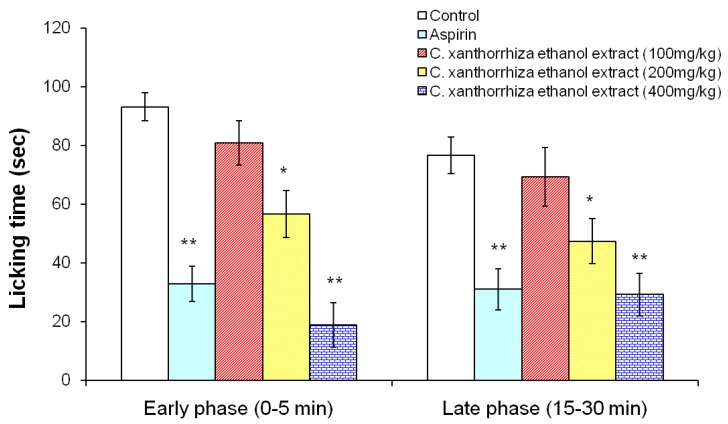
Effects of the standardized ethanolic extracts of *Curcuma xanthorrhiza* and aspirin (100 mg/kg) on formalin induced pain in rats 30 min before subplantar injection of 2.5% formalin (0.05 mL) on the hindpaw of rats. 100, 200 and 400 mg/kg represent the dose of *Curcuma xanthorrhiza*. Data are mean ± SEM values for the paw licking time measured in early phase (0–5 min) and late phase (15–30 min). *p < 0.05 compared to the control group, treated with cosolvent (ANOVA, Dunnett’s test).

The formalin test is a pain model which assesses the way an animal responds to continuous pain generated by injured tissue [15]. The formalin test has a distinctive biphasic peripheral nociceptive response termed as the early and late phases. The early phase or tonic pain response corresponds to the neurogenic phase which is directly stimulated in the paw with the release of substance P. The late phase refers to the inflammation pain response involving the release of histamine, serotonin, bradykinin and prostaglandin [16]. The standardized ethanolic extract of *Curcuma xanthorrhiza* has revealed significant (p < 0.05) analgesic effects on formalin induced pain in both early (0–5 min) and late phases (15–30 min), as shown in Figure 2. The treated groups at 200 and 400 mg/kg showed significant reduction in the licking of paw in the early phase (neurogenic pain) and late phase (inflammatory pain) respectively. Aspirin as a peripheral analgesic significantly reduced the licking of paws in both phases compared to the control. In this study, standardized *Curcuma xanthorrhiza* ethanolic extract reduced the neurogenic pain (early phase) caused by formalin. As it was previously reported, some neuropathic pains can be caused by central neurogenic mechanism due to the ascending nociceptive pathway such as spinothalamic tract (STT) [17]. In contrast, thermal pain in hot plate and tail flick tests is mediated by supraspinal and spinal nociceptive pathways respectively, which are dissimilar to the neurogenic mechanism [11,12]. Further, xanthorrhizol in *Curcuma xanthorrhiza* ethanolic extract has been reported to possess anti-inflammatory effect based on the previous study by Lim *et al* [7]. This suggests that the presence of xanthorrhizol in the standardized ethanolic extract of *Curcuma xanthorrhiza* may partly contribute to the anti-inflammatory effects observed in the late phase of formalin test. 

## 3. Experimental

### 3.1. Materials

All chemicals and standards were obtained from Sigma Aldrich, USA. Xanthorrhizol (standard) was purchased from Alexis, USA. Aspirin (300 mg/kg) and morphine (5 mg/kg) were prepared by dissolving in saline solution (0.9% NaCl). Formalin (2.5%) was diluted with distilled water. The extract was suspended in cosolvent solution of propylene glycol -Tween 80-distilled water (4:1:4) and referred as vehicle at the time of the oral administration for doses at 100, 200 and 400 mg/kg body weight.

### 3.2. Plant material identification

*Curcuma xanthorrhiza* plants were obtained from the Johor Plantation, Malaysia. A voucher specimen (11022) was authenticated and deposited at the Herbarium Unit of the School of Biological Sciences, Universiti Sains Malaysia.

### 3.3. Preparation of the extracts

The rhizome portion of *Curcuma xanthorrhiza* was purchased in powder form from Chemical Engineering Pilot Plant (CEPP), UTM, Skudai, Johor, Malaysia. The coarsely powdered material (800 g) was macerated with absolute ethanol (8 L) for 72 h with occasional shaking. The maceration was repeated thrice. The extract was filtered and concentrated at reduced pressure on rotary evaporator resulting in dark yellow colored semisolid mass (yield 5.2%).

### 3.4. Animals

Male Sprague-Dawley rats (130–180 g) and female ICR mice (20–25 g) were obtained from the Animal House, Universiti Sains Malaysia. The animals were acclimatized to laboratory conditions for seven days prior to the experiments. Five rats were housed per polycarbonate cage, with free access to food (normal laboratory chow, Gold Coin) and tap water *ad libitum*. The animals were maintained at room temperature under a light/dark cycle of 12 h. All experiments were performed between 9.00 a.m. to 2.00 p.m. in order to prevent any confrontation with circadian rhythm. Experimental protocols and procedures employed in this study were approved by the Animal Ethics Committee of the Universiti Sains Malaysia with the reference number USM/PPSF/50(072) Jld 2.

### 3.5. Standardization of Curcuma xanthorrhiza ethanolic extract

The xanthorrhizol amount in the ethanolic extract of *Curcuma xanthorrhiza* was determined using a gas chromatography mass-spectrometry (GC/MS) system which consisted of an Agilent 6890 gas chromatograph (column) coupled with an Agilent 5973 mass spectrometry. Separation was performed with HP-5MS capillary column (30 m × 0.25 mm × 0.25 µm). The injector was set at 280 °C and GC was performed in the splitless mode with 1 min splitless time. The oven temperature was initiated at 70 °C for 2 min and increased to 250 °C, held for 11 min at 20 °C ramp/min. The flow rate of the carrier gas (helium) was maintained at 0.5 mL/min. The septum purge was set at 1 mL/min. The mass spectrometer was operated with ionization in the electron impact (EI) mode using 70 eV (ionization energy). Standard xanthorrhizol was injected at concentrations ranging from 1–50 µg/mL for a calibration curve. The volume of *Curcuma xanthorrhiza* ethanolic extract injected was 1 µg (3 mg of extract in 1 mL of absolute ethanol). Quantification was done in the single-ion monitoring (SIM) mode. Identifications were made by comparison with the NIST library GC-MS system and by their retention indexes. 

### 3.6. Phytochemical screening

Phytochemical screening of the standardized *Curcuma xanthorrhiza* ethanolic extract was performed according to the procedures described by Sofowora [18], Harborne [19] and Siddique and Ali [20]. This experiment was carried out to detect the presence of distinct constituents such as reducing sugars, saponins, alkaloids, flavonoids, tannins, steroids, terpenoids, cardiac glycosides and anthraquinones.

### 3.7. Acute toxicity test

Mice were divided into control and test groups (*n* = 5). The test groups received a single oral dose of standardized *Curcuma xanthorrhiza* ethanolic extract at 300 mg/kg, 2000 mg/kg and 5000 mg/kg respectively. After treatment, mice were observed for 30 min and thereafter for 14 days in order to notice any signs of toxicity, mortality or behavioral changes [21].

### 3.8. Antinociceptive activity

#### 3.8.1. Hot plate test

The hot plate test was conducted in accordance to the method described by Woolfe and MacDonald [11] with slight modifications. The hot plate test was assessed by the Incremental Hot plate (IITC Life Sciences). The hot plate temperature was maintained at 55 ± 1 °C. Prior to treatment, only rats that showed response within 18 sec were selected for this study. Rats (five per group) were administered with the standardized *Curcuma xanthorrhiza* ethanolic extract [100, 200 and 400 mg/kg, per oral (p.o)], aspirin (300 mg/kg, p.o), morphine [5 mg/kg, subcutaneous (s.c)] and vehicle (cosolvent) respectively. Latency time of animal’s response to heat induced pain such as licking of paw or jumping was measured for every 15 min over a 60 min period. Observation started after 30 min of administration of the test substances except for morphine which was 15 min after administration. The cut-off time was set as 45 s to prevent tissue damage. 

#### 3.8.2. Tail flick test

The tail flick was assessed by the analgesiometer (IITC Life Sciences) and using the procedures as stated by D’Amour and Smith [12] with some modifications. Rat’s response to this focused heat stimulus for example flicking or removing their inflicted tail was referred as latency time (s). Prior to treatment, a sensitivity test was conducted and rats that did not attempt to withdraw tail within 4 sec were discarded. The selected rats were allotted into control and test groups (*n* = 5). Similar dose regimens were provided as described in hot plate test. The cut-off time was fixed as 10 s. After 30 min administration of the test substances and 15 min for morphine, the latency time was measured for every 15 min intervals for 1 h.

#### 3.8.3. Formalin test

The method employed was slightly amended to that described previously by Dobuisson *et al.* [15]. Pain was induced by injecting 0.05 mL of formalin (2.5%) subcutaneously in the sub plantar of the right hind paw of the rats. Rats (five per group) received standardized *Curcuma xanthorrhiza* ethanolic extract (100, 200 and 400 mg/kg, p.o), aspirin (100 mg/kg) and vehicle (cosolvent, p.o) 30 min prior to the formalin injection. These rats were placed in separate cages for the observation. The time (s) spent for licking of the injected paw was considered as indicative of pain. Nociceptive responses were measured for first 5 min (early phase) and 15–30 min (late phase) after formalin injection. 

### 3.9. Statistical analysis

The statistical analysis was performed by one-way ANOVA followed by Dunnett’s multiple comparison tests in SigmaStat® version 3.5 Software. The results were expressed as mean ± S.E.M to show differences in groups. The differences are considered significant when P < 0.05.

## 4. Conclusions

The experimental evidence acquired in this study implied that standardized ethanolic extract of *Curcuma xanthorrhiza* is non-toxic and possesses peripheral antinociceptive effects by blocking the inflammation pain response. The standardized ethanolic extract of *Curcuma xanthorrhiza* also exhibited a central antinociceptive effect which is associated with the neurogenic pathway and not the opioid pathway. Xanthorrhizol, one of the major constituent quantified in this extract, may contribute to the respective antinociceptive effects. However, further investigation is required to conclusively prove antinociceptive activity on the bioactive compound/s. 
